# Central retinal microvasculature damage is associated with orthostatic hypotension in Parkinson’s disease

**DOI:** 10.1038/s41531-023-00480-6

**Published:** 2023-03-09

**Authors:** Jong Hyeon Ahn, Min Chae Kang, Dongyoung Lee, Jin Whan Cho, Kyung-Ah Park, Jinyoung Youn

**Affiliations:** 1 Department of Neurology, Samsung Medical Center, Sungkyunkwan University School of Medicine, Seoul, Republic of Korea; 2 Neuroscience Center, Samsung Medical Center, Seoul, Republic of Korea; 3 Department of Ophthalmology, Samsung Medical Center, Sungkyunkwan University School of Medicine, Seoul, Republic of Korea

**Keywords:** Parkinson's disease, Neuro-vascular interactions

## Abstract

Orthostatic hypotension (OH) is a common non-motor symptom in Parkinson’s disease (PD). OH can cause cerebral and retinal hypoperfusion and is associated with microvascular damage in PD. Optical coherence tomography angiography (OCTA) is a non-invasive technology that can be used to visualize the retinal microvasculature and detect microvascular damage in PD. In the present study, 51 PD patients (OH+, *n* = 20, 37 eyes; OH−, *n* = 32, 61 eyes) and 51 healthy controls (100 eyes) were evaluated. The Unified Parkinson’s Disease Rating Scale III, Hoehn and Yahr scale, Montreal Cognitive Assessment, levodopa equivalent daily dose, and vascular risk factors, including hypertension, diabetes, and dyslipidemia, were investigated. PD patients underwent a head-up tilt (HUT) test. The PD patients had a lower superficial retinal capillary plexus (SRCP) density in the central region than control patients. The PDOH+ group had lower vessel density in the SRCP of the central region compared with the control group and lower vessel density in the DRCP of the central region than the PDOH− and control groups. The changes in systolic and diastolic blood pressure during the HUT test in PD patients showed a negative correlation with the vessel density in the DRCP central region. The presence of OH was a critical factor associated with central microvasculature damage in PD. These findings indicate that OCTA can be a useful and non-invasive tool for detecting microvasculature damage in PD patients.

## Introduction

Orthostatic hypotension (OH) is a common non-motor symptom in Parkinson’s disease (PD), even in the early stage of the disease^1^. OH in PD is associated with a higher mortality rate, cognitive decline, higher fall risk, and poorer quality of life^2^. OH induces a recurrent episodic cerebral and retinal hypoperfusion and presents with various symptoms such as blurred vision, loss of peripheral vision (gray-out), color changes, and scotomas^3^. The underpinnings of the relationship between OH and clinical outcomes in PD are not well understood; however, a vascular hypothesis has been suggested as a possible pathophysiological mechanism^2^. The fluctuation of systemic blood pressure (BP) and impairment of autoregulation induce microvasculature damage in patients with PD^4^. However, this is difficult to evaluate, as investigating microvasculature damage in vivo is challenging.

Optical coherence tomography angiography (OCTA) is a novel non-invasive technology that can be used to visualize retinal microvasculature. The observation of ocular microcirculation provides an opportunity to evaluate how human circulation responds to stress stimuli^5^. OCTA is useful for detecting microvascular change induced in diabetes and glaucoma, as well as systemic hypotension^5,6^. Microvascular changes have been proposed as a potential contributor to PD, and OCTA has previously been used to detect retinal microvascular changes in PD^7^. PD patients were shown to have a lower macular vessel density than healthy controls, indicating that OCTA parameters can be potential diagnostic biomarkers^8,9^. In contrast, others reported no significant differences in the OCTA parameters of patients with PD compared with healthy controls or patients with essential tremors, and scientific evidence explaining the inconsistent results and pathophysiology of microvascular alterations in PD is lacking^10–12^. In the present study, we hypothesized that OH in PD might be associated with microvasculature damage and that OCTA might be a useful tool for detecting such damage in PD patients.

## Results

The analysis included 51 patients with PD (61 eyes) and 51 healthy controls (100 eyes). The demographics and clinical characteristics of the enrolled participants are described in Table 1. Significant differences in age, sex, vascular risk factors, and spherical equivalent refractive errors (SERs) were not found between the PD patients and controls. Twenty patients (37 eyes) had OH (PDOH+), 31 (61 eyes) did not (PDOH−), the prevalence of OH was 39.2%, and females were more affected. The mean age, sex, and SERs were comparable among the PDOH+, PDOH−, and control groups. Significant differences in the disease duration, Unified Parkinson’s Disease Rating Scale (UPDRS) III, Hoehn and Yahr (H&Y) scale, levodopa equivalent daily dose (LEDD), and Montreal Cognitive Assessment (MoCA) were not observed between the PDOH+ and PDOH− groups. The mean systolic BP (SBP) and diastolic BP (DBP) measured in the supine position were not significantly different between the PDOH+ and PDOH− groups. The mean decreases in SBP from supine to head-up tilt position in the PDOH+ and PDOH− groups was 33.3 and 8.9 mmHg, respectively. The mean DBP decrease from supine to head-up tilt position was also higher in the PDOH+ group than in the PDOH− group (14.2 vs. 3.4 mmHg). The prevalence of supine hypertension was 25.0% in the PDOH+ group and 6.5% in the PDOH− group.

**Table 1:** Demographics and clinical characteristics of the participants.

	PD (*n* = 51, 98 eyes)	Controls (*n* = 51, 100 eyes)	PDOH+ (*n* = 20, 37 eyes)	PDOH− (*n* = 31, 61 eyes)	*p* value^b^	*p* value^c^
Age (years)	65.4 ± 6.3	65.3 ± 6.7	65.3 ± 6.3	65.3 ± 7.1	0.983	>0.999
Sex (male/female)	19/32	19/32	4/16	15/16	>0.999	0.123
Disease duration (months)	51.1 ± 32.8	–	60.3 ± 40.1	45.2 ± 26.1	–	0.109
UPDRS III^a^	12.5 ± 7.6	–	14.5 ± 9.9	11.1 ± 5.5	–	0.123
H&Y scale^a^	1.7 ± 0.5	–	1.8 ± 0.5	1.6 ± 0.5	–	0.451
LEDD (mg)	384.1 ± 228.6	–	406.0 ± 241.8	369.9 ± 222.6	–	0.587
MoCA	26.5 ± 2.0	–	26.8 ± 1.8	26.4 ± 2.1	–	0.667
SER (diopters)^b^	−0.10 ± 1.71	−0.05 ± 1.62	−0.24 ± 1.42	−0.01 ± 1.86	0.868	0.796
HTN (%)	29.4	23.5	30.0	29.0	0.501	0.676
DM (%)	9.8	13.7	0.0	16.1	0.539	0.180
DL (%)	13.7	21.6	10.0	16.1	0.102	0.263
Mean supine SBP (mmHg)			128.8 ± 15.5	128.0 ± 19.5		0.873
Mean supine DBP (mmHg)			69.2 ± 6.7	70.5 ± 11.6		0.867
Mean change SBP (mmHg)			33.3 ± 15.9	8.9 ± 8.4		<0.001
Mean change DBP (mmHg)			14.2 ± 8.0	3.4 ± 3.8		<0.001
Supine HTN (%)	13.7		25.0	6.5		0.060

Data are presented as mean and standard deviation (SD).

*OH* orthostatic hypotension, *UPDRS* Unified Parkinson’s Disease Rating Scale, *H&Y* Hoehn and Yahr, *HTN* hypertension (previously diagnosis), *DM* diabetes mellitus, *DL* dyslipidemia, *LEDD* levodopa equivalent daily dose, *MoCA* Montreal Cognitive Assessment, *SER* spherical equivalent refractive errors, *SBP* systolic blood pressure, *DBP* diastolic blood pressure.

^a^Evaluated at the medication on state.

^b^Comparison of *p* and controls analyzed using the Student’s *t* test or chi-square test.

^c^Comparison of PDOH+, PDOH−, and controls analyzed using the analysis of covariance or chi-square test.

### Comparison of OCT and OCTA parameters between PD patients and healthy controls

The macular retinal thickness and peripapillary nerve fiber layer (pRNFL) thickness were measured using OCT. Significant differences in macular retinal thickness or pRNFL thickness were not found between the PD patients and controls. Macular retinal thickness nor pRNFL thickness differed among the three groups (Table 2).

**Table 2:** Comparison of retinal layer thickness and parafoveal vessel densities in patients with Parkinson’s disease and healthy controls.

	PD (98 eyes)	Controls (100 eyes)	PDOH+ (37 eyes)	PDOH− (61 eyes)	*p* value^a^	*p* value^b^
pRNFL thickness (μm)						
Average	100.1 ± 12.8	101.8 ± 8.2	101.7 ± 14.7	99.1 ± 11.5	>0.999	>0.999
Temporal	74.5 ± 12.2	75.9 ± 11.3	76.4 ± 13.1	73.4 ± 11.6	>0.999	>0.999
Inferior	131.0 ± 21.8	128.7 ± 17.0	134.1 ± 26.1	129.1 ± 18.9	0.390	>0.999
Nasal	69.2 ± 13.4	71.2 ± 15.6	70.4 ± 12.9	68.5 ± 13.7	>0.999	>0.999
Superior	125.5 ± 19.7	126.2 ± 17.5	126 ± 18.3	125.2 ± 20.6	>0.999	>0.999
Total macular thickness (μm)						
Central (1 mm)	262.6 ± 19.9	271.9 ± 18.2	257.3 ± 21.8	265.8 ± 18.0	0.095	0.261
Average (3 mm)	331.7 ± 15.9	337.8 ± 13.3	328 ± 13.4	333.9 ± 16.9	>0.999	>0.999
Temporal (3 mm)	324.8 ± 17.1	330.0 ± 13.6	321.5 ± 13.7	326.8 ± 18.6	>0.999	>0.999
Inferior (3 mm)	331.1 ± 16.5	337.2 ± 13.9	327.7 ± 13.2	333.2 ± 17.9	>0.999	>0.999
Nasal (3 mm)	336.3 ± 16.6	342.4 ± 14.2	331.3 ± 15.1	339.4 ± 16.8	>0.999	>0.999
Superior (3 mm)	334.6 ± 18.3	341.8 ± 13.4	331.8 ± 15.5	336.4 ± 19.8	0.670	>0.999
Average (6 mm)	296.3 ± 14.6	301.6 ± 15.0	296.3 ± 9.9	296.2 ± 16.8	>0.999	>0.999
Temporal (6 mm)	281.5 ± 16.5	287.1 ± 15.0	281.1 ± 14.3	281.7 ± 17.9	>0.999	>0.999
Inferior (6 mm)	293.2 ± 19.2	297.9 ± 26.8	295.8 ± 20	291.6 ± 18.7	>0.999	>0.999
Nasal (6 mm)	306.9 ± 16.0	314.1 ± 14.8	304.1 ± 14.5	308.6 ± 16.7	>0.999	>0.999
Superior (6 mm)	302.7 ± 15.2	307.4 ± 20.4	302.6 ± 12.9	302.7 ± 16.5	>0.999	>0.999
FAZ area (mm^3^)	0.329 ± 0.105	0.274 ± 0.667	0.361 ± 0.130	0.309 ± 0.807	0.017	0.049^c^
SRCP (%)						
Average	47.6 ± 2.1	46.4 ± 2.5	48.1 ± 2.2	47.4 ± 2.1	0.425	0.620
Central	17.8 ± 4.5	19.6 ± 3.3	16.5 ± 4.6	18.6 ± 4.3	0.007	0.008^c^
Temporal	46.6 ± 3.7	45.9 ± 3.0	47.2 ± 3.3	46.3 ± 3.9	>0.999	>0.999
Inferior	48.7 ± 3.9	46.8 ± 4.1	49.4 ± 4.2	48.2 ± 3.7	0.661	0.544
Nasal	46.4 ± 2.7	45.1 ± 3.2	46.3 ± 2.8	46.5 ± 2.6	0.424	0.674
Superior	48.9 ± 2.8	47.7 ± 3.5	49.4 ± 2.6	48.5 ± 2.9	0.526	0.444
DRCP (%)						
Average	49.2 ± 3.0	48.0 ± 2.5	49.8 ± 2.9	48.9 ± 3.1	0.166	0.356
Central	14.8 ± 4.6	16.0 ± 4.8	12.5 ± 3.6	16.2 ± 4.6	0.296	<0.001^c,d^
Temporal	46.5 ± 4.1	46.6 ± 3.2	46.7 ± 3.7	46.4 ± 4.3	0.070	>0.999
Inferior	51.7 ± 5.8	49.5 ± 4.5	52.9 ± 6.2	51.0 ± 5.5	>0.999	0.094
Nasal	48.0 ± 4.1	47.1 ± 3.7	48.2 ± 3.5	48.0 ± 4.4	>0.999	>0.999
Superior	50.7 ± 3.6	49.0 ± 3.8	51.3 ± 3.3	50.3 ± 3.7	0.076	0.141

Data are presented as mean and standard deviation. The Bonferroni correction was performed for multiple comparison.

*PD* Parkinson’s disease, *OH* orthostatic hypotension, *PDOH+* Parkinson’s disease patients with orthostatic hypotension, *PDOH−* Parkinson’s disease patients without orthostatic hypotension, *OCT* optical coherence tomography, *SRCP* superficial retinal capillary plexus, *DRCP* deep retinal capillary plexus.

^a^PD vs. control analyzed using the generalized estimating equation (GEE) analysis.

^b^PDOH+, PDOH− and controls analyzed using the generalized estimating equation (GEE) analysis.

^c^Statistically significant between the PDOH+ and control group.

^d^Statistically significant between the PDOH+ and PDOH− groups.

**Fig. 1:**
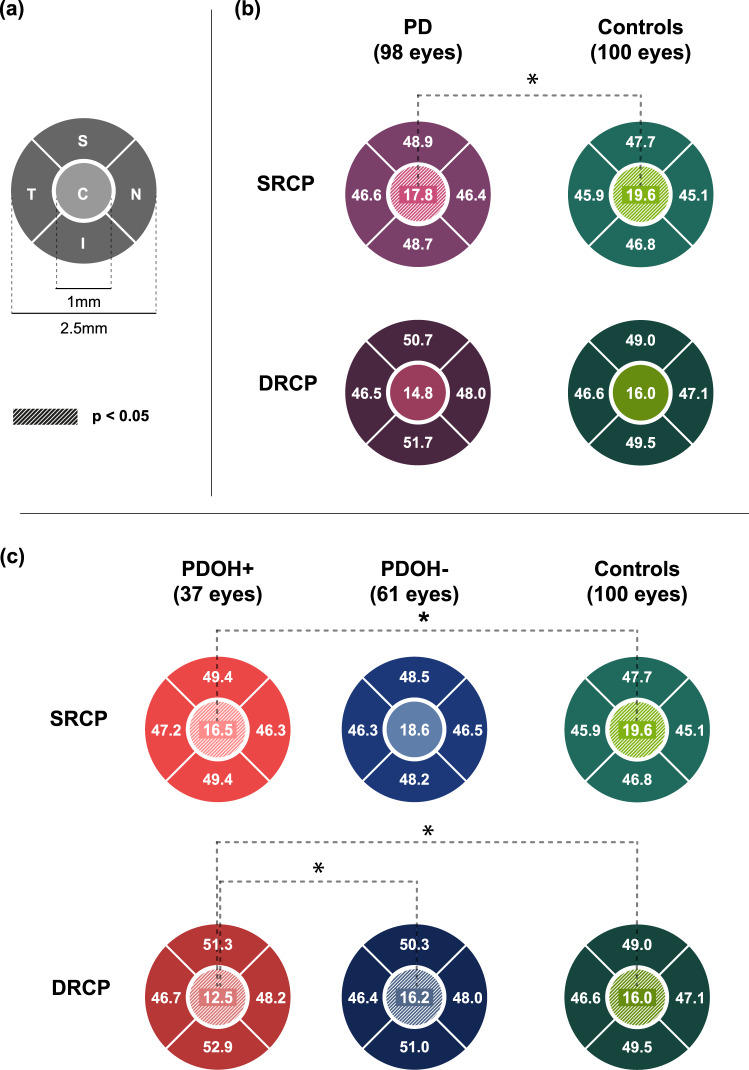
Comparisons of OCTA parameters between patients with Parkinson’s disease and healthy controls. Schematic diagram and size of OCTA subregions (**a**). The Parkinson’s disease (PD) patients had lower superficial retinal capillary plexus (SRCP) vessel density in the central region than controls (**b**). The PD patients with orthostatic hypotension (PDOH+ group) showed a lower vessel density in the DRCP central region than the PD patients without orthostatic hypotension (PDOH−) and the control group (**c**). The SRCP vessel density in the central region in the PDOH+ group was lower than that in the control group but was non-significant compared with the PDOH− group (**c**). The asterisk indicates statistically significant. The hatched area represents statistical significance. The numbers represent the mean value of the vessel density. C center, T temporal quadrant, I inferior quadrant, N nasal quadrant, S superior quadrant.

The OCTA parameters of the participants are described in Table 2. The foveal avascular zone (FAZ) area was larger in the PD group than in the control group. The PD patients had a lower superficial retinal capillary plexus (SRCP) vessel density in the central region than the controls. In contrast, significant differences were not observed between the PD patients and controls in the temporal, inferior, nasal, or superior vessel densities in the SRCP and DRCP (Fig. 1b). To investigate the differences based on the presence or absence of OH in PD, we compared the OCTA parameters among the PDOH+, PDOH−, and control groups. The FAZ area was larger in the PDOH+ group than in the control group. The PDOH+ group showed a lower vessel density in the DRCP of the central region than the PDOH− and control groups. The central SRCP vessel density in the PDOH+ group was lower than in the control group but was not significant when compared with the PDOH− group. In contrast, none of the OCTA parameters showed significant differences between the PDOH− and control groups (Fig. 1c).

### Relationship between the changes of blood pressure and central retinal vessel densities

**Fig. 2:**
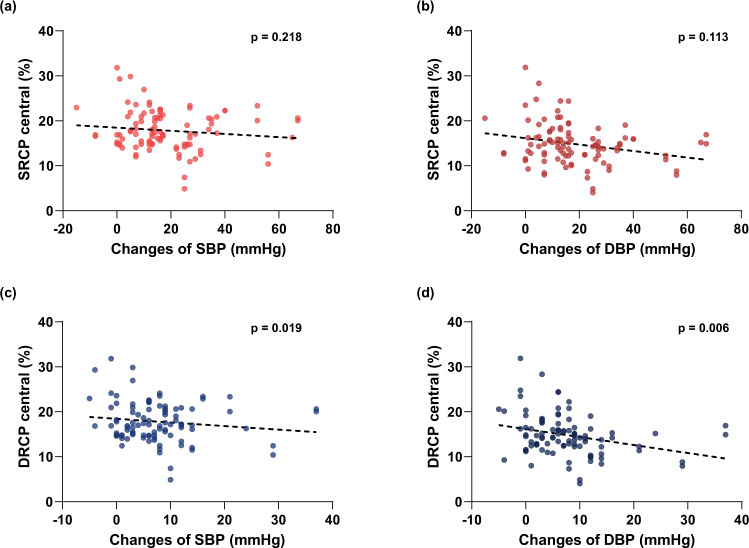
Correlations between the OCTA parameters and changes in blood pressure. The superficial retinal capillary plexus (DRCP) vessel density in the central region did not have a significant relationship with the changes in systolic blood pressure (SBP) and diastolic blood pressure (DBP) (**a**, **b**). The deep retinal capillary plexus (DRCP) vessel density in the central region had a negative correlation with changes in SBP and DBP (**c**, **d**).

To investigate the relationship between the central SRCP and central DRCP vessel densities and changes in blood pressure, we performed linear mixed model analysis. The results indicate that the central SRCP vessel density was not associated with changes in SBP (*β* = −0.054, standard error [SE] = 0.028, *p* = 0.218) or DBP (*β* = −0.128, SE = 0.058, *p* = 0.218). Central DRCP vessel density was associated with changes in SBP (*β* = −0.077, SE = 0.058, *p* = 0.019) and DBP (*β* = −0.180, SE = 0.057, *p* = 0.006) (Table 3 and Fig. 2).

**Table 3:** Results of the linear mixed model analysis.

		Beta coefficient (SE)	95% CI	*p* value^a^
SRCP central	ΔSBP	−0.054 (0.028)	−0.108, 0.001	0.218
	ΔDBP	−0.128 (0.058)	−0.014, −0.014	0.113
DRCP central	ΔSBP	−0.077 (0.027)	−0.130, −0.023	0.019
	ΔDBP	−0.180 (0.057)	−0.291, −0.069	0.006

Linear mixed model after adjustment for age, sex, disease duration, side of the eyes, spherical equivalent refractive errors, supine hypertension, hypertension, diabetes, and dyslipidemia.

*SE* standard error, *SRCP* superficial retinal capillary plexus, *DRCP* deep retinal capillary plexus, *ΔSBP* change of systolic blood pressure during the head-up tilt test, *ΔDBP* change of diastolic blood pressure during the head-up tilt test.

^a^*p* values were corrected for multiple comparisons using the Bonferroni correction.

## Discussion

This is the first study in which the change in OCTA parameters was investigated based on the presence of OH in PD patients. PD patients had significantly lower central retinal vessel densities than healthy controls, and the difference remained significant when comparing the PDOH+ group with the control group but was not significant in the PDOH− group. Furthermore, the amount of SBP and DBP changes correlated with the vessel density in the DRCP central area. These results indicate the central retinal vessel densities measured using OCTA are significantly associated with the presence of OH in PD patients.

Growing evidence shows that alterations of vasculature contribute to the development and aggravation of neurodegenerative diseases, including PD^7,13,14^. OCTA is an emerging non-invasive technology used to investigate in vivo retinal microvascular alterations in various neurological disorders^15^. Robbinson and colleagues suggested that OCTA parameters, especially the inner ring of the macula, are potential biomarkers for the diagnosis of PD^9^. In recent studies on PD, OCTA parameters were suggested as biomarkers for diagnosis and associated with disease progression and cognitive decline^16,17^. In contrast, Rascuna et al. reported that PD patients showed no significant differences in OCTA parameters in DRCP or SRCP compared with healthy controls^10^. When the OCTA parameters in PD, essential tremor, and healthy controls were compared, differences were not observed among the three groups^11^. These inconsistent results indicate that a range of factors can affect OCTA findings. Based on the results of the present study, the presence of OH is presumed to be a crucial factor associated with OCTA parameters in PD patients. When the PD patients were divided into two groups based on the presence or absence of OH, the PDOH+ group had lower retinal vessel densities in the SRCP and DRCP compared with the PDOH− and control groups (Fig. 2). The results indicated that central retinal microvascular changes could be associated with OH in addition to PD, and OCTA can be useful for their detection. In terms of the area of FAZ, it was larger in the PD group than in the control group. The difference was still significant when comparing the PDOH+ and the control groups, but it was not significant between the PDOH− and the control groups. In PD, several studies reported that there were no differences in FAZ area between patients and controls^9,18–20^, although two studies reported that PD patients had smaller FAZ area than controls^16,21^ Murueta-Goyena et al. suggested that smaller FAZ area was a distinguishable feature of PD patients, and it was negatively correlated with cognitive function^21^. Xu et al. suggested that foveal dopaminergic neuronal damage is an underlying mechanism of the reduction in FAZ area^16^. The patients included in the present study had normal cognitive function, milder motor symptoms, and lower LEDD compared with those of the previous studies^21^. The discrepancy between study results might be due to differences in baseline characteristics of the included patients, such as age distribution, PD phenotypes, disease severity, disease duration, cognitive function, and concomitant neurological or systemic conditions.

OH is a common non-motor symptom in PD that induces recurrent episodic cerebral and retinal hypoperfusion^3^. Recurrent episodic cerebral hypoperfusion may result in cerebral microvascular and white matter damage. PD patients with OH had increased white matter hyperintensities on brain magnetic resonance imaging (MRI) compared with the patients without OH^22^, and chronic cerebral hypoperfusion was associated with microvascular pathology in the brains of PD model mice^23^. In this context, OH may induce retinal microvascular damage in addition to cerebral microvascular damage. In terms of retinal vessels, microvascular damage may be associated with hypoperfusion induced by carotid artery stenosis as well as systemic hypoperfusion. For example, changes in OCTA metrics are associated with intradialytic hypotension episodes in chronic hemodialysis patients^6^. Furthermore, early changes in retinal microvasculature have predictive value regarding the development of systemic vascular disorders^24,25^. However, because a control group with OH and/or supine hypertension was not included in the present study, it remains unknown whether the changes are PD specific. Further studies that include controls with OH, supine hypertension, and patients with other diseases that show OH (drug-induced OH, pure autonomic failure, or multiple system atrophy) can aid in understanding the relationships among fluctuations in blood pressure, PD, and central retinal microvascular damage.

In the present study, significant changes were found in superficial and deep retinal vessel density only in the central macular area, indicating that the central retinal area is predominantly affected in patients with PD. The results are in agreement with previous studies in which the central retinal area was more greatly affected in PD patients^9,12,18,19^, indicating that the retinal vessels in the central area are more vulnerable to hypoperfusion and disease. The FAZ area, which is a central area highly sensitive to ischemic events such as diabetes^26^ and retinal vascular obstruction^27^, was increased in the PD group, particularly in the PDOH+ group. In addition, thinning of the macular inner retinal layers, an emerging biomarker of PD, reportedly occurs mainly in the parafoveal area in the early stages of PD^28^. This result supports the assumption that the central macular area is the most affected or vulnerable retinal area in PD patients^28^. However, there is a lack of evidence to explain the pathomechanism, and further research is needed to corroborate this hypothesis.

In addition, the increased reduction in SBP and DBP during the head-up tilt (HUT) test was significantly associated with lower vessel density in DRCP. The retinal artery consists of two parallel vascular networks. The superficial vascular plexus consists of approximately 75-µm diameter vessels supplied by the central retinal artery and smaller deep capillary plexuses (20-µm diameter) supplied by vertical anastomoses from the superficial vascular plexus^29,30^. The smaller vessels in DRCP may be more sensitive to hypoperfusion induced by OH than SRCP^29^. The relationship found between SBP, DBP, and retinal vessel density is in agreement with previous studies in which the association between BP and hypoperfusion or neuronal damage was analyzed. In previous studies, lower SBP and DBP were reportedly associated with the progression of normal tension glaucoma^31^, exacerbation of cerebral hypoperfusion and brain atrophy^32^, and leukoaraiosis^33^, and lower DBP also contributed to the development of dementia^34–36^. A similar mechanism could occur in the retina. In summary, both the SRCP and DRCP are affected by OH in PD. The DRCP is more likely to be affected, and the reduction of SBP and DBP might play an important role in damage to the microvasculature of the central retina in PD patients.

Increasing evidence shows the importance of the retina as a potential biomarker of early diagnosis and prognostication in PD^28^. In recent studies, parafoveal inner retinal change was shown to be detected in the early stages of PD, followed by progressive atrophy of pRNFL and macula over time^28^. In the present study, although the intraocular vessel density was significantly reduced in PD patients compared with controls, the pRNFL thickness and macular retinal thickness, which represent the degree of neuroaxonal damage, did not show a significant difference between PD patients and controls, even in the PDOH+ group. Rascuna et al. showed a positive correlation between retinal thicknesses (RNFL, ganglion cell layer, and inner plexiform layer) and microvascular density in the foveal region, and they suggested that microvascular change and macular atrophy reciprocally interact in PD^37^. In contrast, Robbinson and colleagues reported microvascular change without macular atrophy in PD patients^9^, suggesting microvascular change could be present prior to macular atrophy. Although macular atrophy is one of the most important potential causes of microvascular change, the results of the present study suggest that retinal microvascular dysfunction may occur primarily in PD rather than secondary to macular atrophy. Further large-scale studies on detailed retinal structures in various stages of the disease are needed to clarify the temporal relationship and precise mechanisms of early retinal and microvascular changes in PD.

The present study has several limitations. First, the HUT test was not performed in the control group; therefore, healthy controls with OH cannot be omitted, but healthy controls who had any neurological signs including dizziness, headache, or orthostatic symptoms were excluded. Second, various vascular risk factors were investigated in this study; however, carotid artery stenosis, obesity, and smoking history were not assessed. Third, the HUT test and OCTA were not performed on the same day, which might have influenced the results. Fourth, vessel density was measured using OCTA, in which the angiographic signal was based on movement. However, many other factors, such as blood flow velocity, morphology, and alterations in the vascular endothelial barrier, can compromise the measurement of perfusion. Therefore, false-positive findings cannot be excluded due to technical and methodological issues. Finally, the OCTA measurements in this study included large blood vessels. The changes in vessel density found in the present study were, therefore, a combination of changes in both the microvasculature and major vessels. The lack of statistical significance in the difference in parafoveal vessel densities between groups in this study could be attributed to those methodological limitations.

Recent research has demonstrated a strong correlation between the retina and PD, and there is growing interest in the role of retinal microvascular changes as a potential biomarker for the development and progression of PD. Despite its importance, in vivo examination of microvascular damage in PD patients is very limited. In the present study, the microvasculature damage in PD patients based on the presence of OH was investigated, and OH was a potentially critical factor associated with central retinal microvascular damage in PD. The results showed that central retinal microvascular damage measured using OCTA occurs prior to the development of macular thinning and pRNFL. Based on the results, OCTA can be a useful non-invasive method for detecting central retinal microvascular damage in PD patients.

## Methods

### Participants and clinical assessment

Participants were recruited from the movement disorder clinic of the Samsung Medical Center. The Institutional Review Board of Samsung Medical Center approved this study, and all subjects provided written informed consent. Patients were enrolled if they were diagnosed with PD based on the United Kingdom Brain Bank Criteria for PD^38^. Patients with any of the following conditions were excluded: any neurologic disorder other than PD, systemic vasculitis, cardiovascular disease, musculoskeletal disease, end-stage renal disease, peripheral nervous system autonomic failure (diabetic neuropathy, Guillain-Barre syndrome, amyloid neuropathy, surgical sympathectomy, and pheochromocytoma, etc.), ocular pathology that could affect OCTA measurements (glaucoma, a refractive error >+6.0 diopters of spherical equivalent or <−6.0 diopters of spherical equivalent, astigmatism ≥ 3.0 diopters, epiretinal membrane, age-related macular degeneration, diabetic retinopathy, hypertensive retinopathy, retinal artery/vein occlusion, or optic neuropathy) or previous retinal surgery. Exact age- and sex-matched controls were recruited. The healthy controls were required to have normal visual acuity, normal intraocular pressure ≤21 mm Hg, and normal optic discs. The same exclusion criteria were applied to healthy controls and PD patients. Demographic and clinical data, including age, sex, and comorbid vascular risk factors (hypertension, diabetes mellitus, dyslipidemia), were collected for all enrolled participants. The UPDRS III^39^, H&Y scale^40^, LEDD^41^, and MoCA^42^ were investigated in all enrolled PD patients.

### OCT and OCTA

**Fig. 3:**
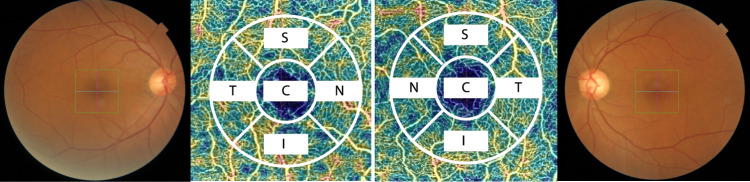
Representative fundus photography and OCTA images of the macular area in a healthy control. Macular OCTA measurement was obtained by automatically applying an Early Treatment Diabetic Retinopathy Study (ETDRS) grid overlay containing the two inner rings of the ETDRS grid pattern to the fovea, which yielded the vessel density in each layer. C center, T temporal quadrant, I inferior quadrant, N nasal quadrant, S superior quadrant.

All included patients and healthy subjects underwent spectral-domain OCT (SD-OCT; Spectralis, Heidelberg Engineering, Heidelberg, Germany) that provided 40,000 A-scans per second with 7-μm optical and 3.5-μm digital axial resolution. An internal fixation target was used, and the patient’s other eye was covered during scanning. OCT peripapillary RNFL circular scans centered on the optic disc of each patient were obtained. In addition, macular thickness measurements in the central 1-mm area and in each quadrant in the 3- and 6-mm areas were obtained. Swept-source OCT (DRI OCT Triton Plus; Topcon Corporation, Tokyo, Japan) coupled with non-invasive OCTA technology was also completed for all PD patients and healthy subjects. The details have been previously described^43,44^. The SRCP slab was automatically segmented from 3 µm under the internal limiting membrane (ILM) to 15 µm below the IPL, and the DRCP slab was automatically segmented from 15 to 70 µm under the IPL following a formerly corroborated method by Park et al.^45^. The radial peripapillary capillary (RPC) segment ranged from the ILM to the posterior boundary of the RNFL. Vessel density was determined as the percentage of the total area occupied by vessels and microvasculature, quantitatively expressed as color-coded vessels in a localized region that was obtained by automatically applying an Early Treatment Diabetic Retinopathy Study (ETDRS) grid overlay containing the two inner rings of the ETDRS grid pattern to the fovea, which yielded a calculation of the density in each layer. The parafoveal ring divided the macular region into the temporal, nasal, inferior, and superior sections (Fig. 3). All participants completed both OCT and OCTA imaging within 1 day. The software generated TopQ image quality values for each OCTA scan and vessel density measurement. To assess scan quality, we included the scan images based on the quality assessment criteria suggested by Fenner et al.^46^. Expert graders reviewed and verified all images (D.L. and K.-A.P.). In patients with PD and healthy controls, bilateral eyes were analyzed except OCTA images, which are difficult to analyze due to motion artifacts or incomplete acquisitions.

### Head-up tilt test

Participants with PD underwent the HUT test. The patients discontinued medications that could affect the HUT test results at least 24 h before the test. In addition, the participants were prohibited from smoking and drinking beverages containing caffeine on the day of the test. The electrode and BP cuff were attached to the patient, and BP was continuously recorded using Finometer 1 (FMS, Amsterdam, the Netherlands) after a period of 5 min of supine rest. OH was defined as a reduction of at least 20 mmHg in SBP or a 10 mmHg fall in DBP within 3 min of HUT testing^47^. In patients with supine hypertension, a reduction in SBP of 30 mm Hg was applied^48^.

### Statistical analysis

The normality of the data was evaluated using the Shapiro–Wilk test. Clinical and demographic features were presented using mean and standard deviation (SD). Differences among the three groups were determined using the Student’s *t* test, analysis of variance (ANOVA), or chi-square test. The OCTA and OCT parameters of the participants were compared using a generalized estimating equation (GEE) analysis with an exchangeable correlation structure to account for the inclusion of both eyes from the same individual. The following variables were also included as confounding factors for the GEE: age, sex, SER, supine hypertension, hypertension, diabetes, and dyslipidemia. Linear mixed model analysis was performed to investigate the association between the OCTA parameters and changes in blood pressure after adjustment for age, sex, side of the eye, disease duration, SER, and vascular risk factors. Four models were established for two dependent variables (central vessel density of the SRCP or central vessel density of the DRCP) and two independent variables (changes in SBP or DBP during the HUT test) because of the collinearity between the variables. Bonferroni correction was applied for multiple comparisons. Results were considered significant if the *p* value was ≤0.05. Statistical analysis was performed with IBM SPSS (version 28.0; IBM Inc., USA) software for Windows.

## Data Availability

The data presented in this work are available on request from the corresponding authors.
